# The PSD-95 inhibitor NA-1 is delivered to the brain upon nasal administration with uptake into the olfactory bulb improved by co-administration with the cell-penetrating peptides lowPro and Tat

**DOI:** 10.1007/s13346-025-01842-8

**Published:** 2025-04-03

**Authors:** Solveig Elle Schmidt, Gunhild Joensen, Camilla Sandbjerg, Maria Thaysen, Bente Gammelgaard, Katharina Schindowski, Mie Kristensen

**Affiliations:** 1 Department of Pharmacy, University of Copenhagen, Universitetsparken 2, Copenhagen Ø, DK-2100 Denmark; 2 Institute of Applied Biotechnology, Biberach University of Applied Sciences, Hubertus-Liebrecht Strasse 35, DE-88400 Biberach/Riss, Germany

**Keywords:** PSD-95 inhibitor, NA-1, Tat-NR2B9c, Nose-to-brain, Cell penetrating peptide, Primary olfactory cell culture model

## Abstract

Ischemic stroke affects millions of people annually with limited treatment options targeting excitotoxicity, a major cause of cognitive impairment. The PSD-95 inhibitor NA-1 has demonstrated neuroprotective potential, but its efficacy via intravenous administration is hindered by broad systemic distribution, reduced brain exposure, and interaction with thrombolytic agents like alteplase. This study explores the potential of nasal administration as an alternative delivery route to enhance brain uptake and reduce systemic off-target effects of NA-1. A porcine primary olfactory model was exploited to evaluate NA-1 permeability and the impact of co-administration with the cell-penetrating peptides Tat, LowPro, and PenShuf. NA-1 alone permeated the model to a greater extent than a similar sized model dextran compound, with PenShuf improving NA-1 permeability but compromising barrier integrity in vitro. In vivo, nasal administration to mice achieved brain uptake of NA-1, particularly in the olfactory bulb, with co-administration of Tat and LowPro enhancing olfactory bulb delivery. Compared to intravenously administered NA-1, nasal delivery resulted in significantly lower off-target tissue distribution. These findings highlight nasal administration as a qualified alternative for NA-1 delivery, with potential to bypass the limitations of intravenous administration and enable concurrent use with alteplase during acute ischemic stroke.

## Graphical Abstract

**Figure:**
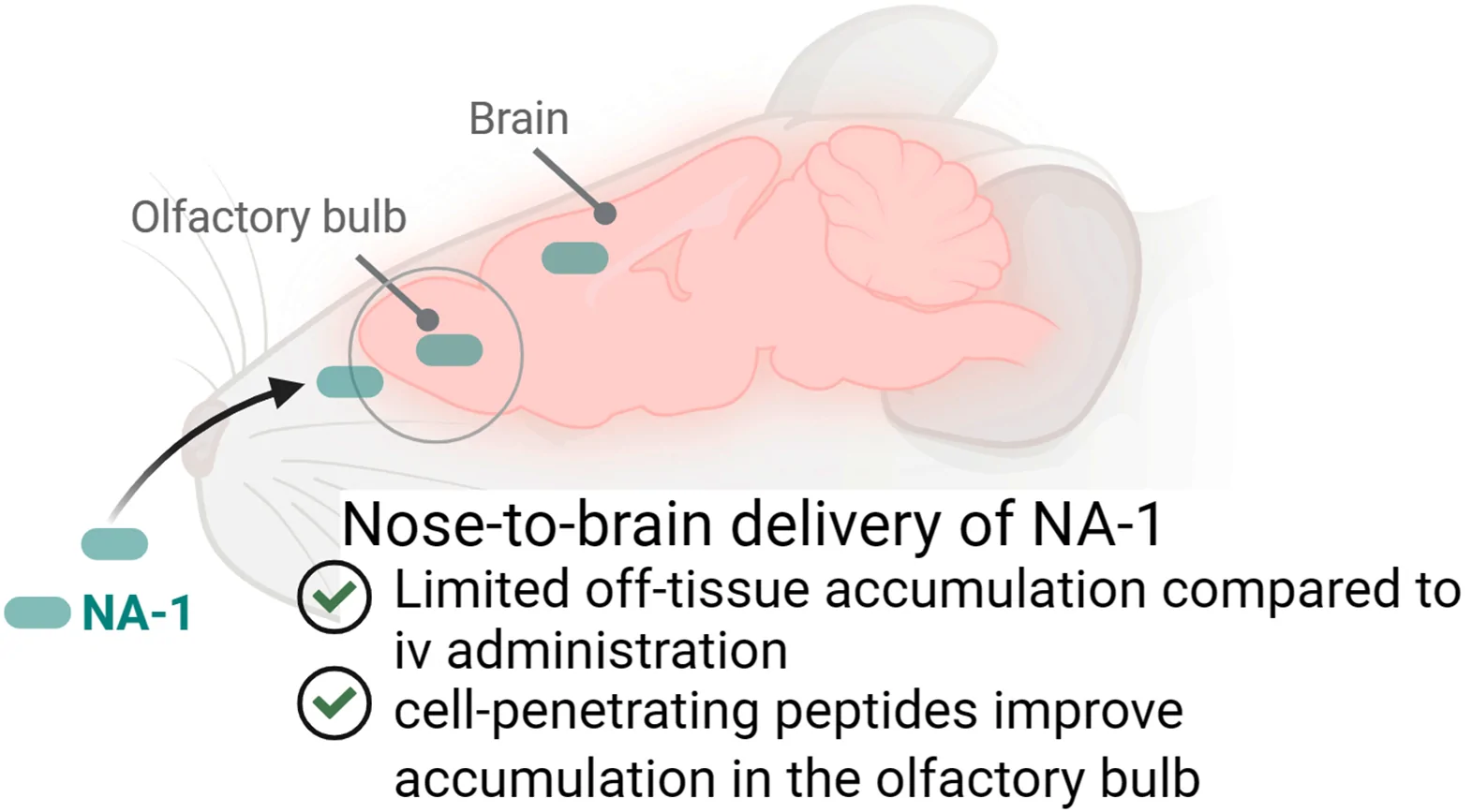

## Introduction

Every year millions of people experience an ischemic stroke [1]. Currently, the only treatment option is reperfusion through thrombectomy or thrombolysis, whereas we completely lack drugs to dampen the ischemic stroke-associated excitotoxicity, leading to impaired patient cognition, which negatively affects life quality. The PSD-95 inhibitor NA-1 (also known as nerinetide) [2] shows promising results in the treatment of ischemic stroke, as evident by pre-clinical studies (e.g [3, 4]). as well as clinical trials advancing through phase 3 (namely ESCAPE-NA1, ESCAPE-NEXT, and FRONTIER). However, NA-1 revealed some limitations upon conventional intravenous administration, since its otherwise neuroprotective effect was abolished in patients receiving the thrombolytic agent alteplase (standard care) to dissolve the blood clot for reperfusion [5]. In addition, NA-1 tends to broadly distribute to various organs upon systemic circulation [6, 7], thereby limiting its exposure to the brain as well as increasing the risk for potential adverse side-effects.

NA-1 is a 20-mer peptide comprised by a therapeutic moiety (KLSSIESDV), which is N-terminally conjugated to the cell-penetrating peptide Tat (YGRKKRRQRRR) in order to facilitate delivery of the former into neurons for target engagement. To improve plasma stability of NA-1, the Tat moiety was more recently synthesized with D-amino acids, which are more resilient to enzymatic breakdown when compared to their natural L-counterparts [8]. To improve brain NA-1 exposure, nasal administration may serve as an alternative to conventional intravenous administration, since the olfactory mucosa in the upper nasal cavity offers a potential direct route into the brain, whereby the otherwise restrictive blood-brain barrier is by-passed and broad off-target biodistribution may be reduced [9]. Successful nose-to-brain delivery has previously been demonstrated for peptide and protein-based drugs including e.g. insulin [10] and an anti-Nogo-A antibody [11]. Studies have moreover suggested that co-administration with cell-penetrating peptides may boost nose-to-brain delivery of a cargo protein such as insulin [12] or bovine serum albumin [13]. We therefore, hypothesized that administration of NA-1 to the upper nasal cavity in mice will result in brain delivery of NA-1 and limited off-target biodistribution when compared to intravenously administered NA-1. Moreover, we investigated whether co-administration with the cell-penetrating peptides Tat (YGRKKRRQRRR), LowPro (VSRRRRRRGGRRRR), or PenShuf (RWFKIQMQIRRWKNKK) improved NA-1 permeation across a primary porcine nasal olfactory cell culture model [14] as well as uptake into the olfactory bulb and the brain upon nasal administration to mice.

## Materials and methods

### Materials

Dulbecco’s Modified Eagle Medium (DMEM) F-12 w/o L-glutamine, w/o 4-(2-hydroxyethyl)-1- piparazineethane sulfonic acid (HEPES), w/o glucose was purchased from BioWest (Nuaillé, France). Minimum essential medium (MEM) w/o phenol red, MEM non-essential amino acids solution and Earle’s balanced salt solution were purchased form Thermo Fisher Scientific (Dreieich, Germany). Fetal bovine serum (FBS) was purchased from Biochrome (Berlin, Germany) or Thermo Fisher Scientific (Dreieich, Germany). Rat tail collagen type I (3 mg/mL) was purchased from PRIMACYT Cell Culture Technology GmbH (Schwerin, Germany). Bovine serum albumin (BSA) (Albumin fraction V), gentamycin sulphate, kanamycin sulphate, and ROTI^®^ Fair phosphate buffered saline (PBS) pH 7.4 tablets were purchased from Carl Roth GmbH (Karlsruhe, Germany). Penicillin-streptomycin (100x) was obtained from AppliChem GmbH (Darmstadt, Germany) or Merck Life Sciences A/S (Søborg, Denmark) Trypsin-Ethylenediaminetetraacetic acid (EDTA) (10x) was purchased from Biochrome (Berlin, Germany) or Merck Life Sciences A/S (Søborg, Denmark). Octenisept^®^ was obtained from Schülke & Mayr GmbH (Norderstedt, Germany) and Debris Removal Solution was purchased from Miltenyi Biotech (Bergisch Gladbach, Germany). Cell culture flasks and black clear-bottom 96-well plates were purchased from Greiner Bio-One (Fricken hausen, Germany) or Corning (Glendale, AZ, USA). Cellstar Multiwell Culture 24-well Plates and ThinCerts Transwells Inserts (1 µm pore size, transparent) were obtained from Greiner Bio-One (Frickenhausen, Germany). A sodium 3’-[1-(phenylaminocarbonyl-3,4-tetrazolium]-bis(4-methoxy6-nitro) benzene sulfonic acid hydrate (XTT) Cell Profiliation Kit II and cOmplete protease inhibitor tablets were purchased from Roche (Basel, Switzerland). The peptides (> 95% purity) NA-1 (ygrkkrrqrrrM^Se^KLSSIESDV), LowPro (VSRRRRRRGGRRRR) and PenShuf (RWFKIQIRRWKNKK) were custom-made by CASLO ApS (Kgs. Lyngby, Denmark) and Tat (YGRKKRRQRRR) by Biomatik (Cambridge, ON, Canada).

All other reagents were obtained from Merck KGaA (Darmstadt, Germany) or Merck Life Sciences A/S (Søborg, Denmark) unless otherwise stated.

### Isolation and culture of primary porcine olfactory epithelial cells

A primary porcine olfactory cell culture model was established as earlier described by Ladel et al. [14]. Snouts were isolated from 2 to 4 months old pigs immediately after euthanasia. The olfactory mucosa was isolated from the dorsal part of the middle and the superior turbinate and transferred to a T25 flask. The mucosa was first disinfected with Octenisept^®^ and thereafter washed three times with ice-cold PBS. Pronase medium (Table 1) was added and the mucosa was homogenized using a 5 mL serotological pipette before being incubated for 1 h (37 °C, 5% CO_2_, 95% relative humidity) and another round of homogenization. The cell suspension was subjected to centrifugation at 500 rcf and 4 °C for 10 min in a Heraeus Multifuge 3 SR + centrifuge (Thermo Fischer Scientific, Dreieich, Germany), where after the pellet was resuspended in ice-cold PBS and transferred to fresh 15 mL Falcon tubes. The resulting pellet was resuspended in ice-cold PBS before Debris Removal Solution was added and the suspension was overlaid with ice-cold PBS and centrifuged at 3000 g for 10 min at 4 °C. Three phases were formed from which the two top phases were discarded. Ice-cold PBS was added to the bottom phase before being centrifuged at 1000 g for 10 min at 4 °C. The olfactory epithelial cells were resuspended in pre-warmed adhesion medium (Table 1) and transferred to collagen-coated T75-flasks. The olfactory epithelial cells were incubated with adhesion medium for 4 h or overnight, after which they were washed with PBS and the medium was changed to cultivation medium (Table 1). If fibroblasts started to outgrow the olfactory epithelial cells in the T75 flasks, they were removed by trypsin-EDTA (1x) treatment for 4 min at 37 °C, which releases the fibroblasts but not olfactory epithelial cells.

At 80–100% confluency the olfactory epithelial cells were detached from the culturing flasks by a two-step trypsinization procedure. The cells were incubated with trypsin-EDTA (1x) for 4 min at 37 °C, followed by a PBS wash and then incubation with trypsin-EDTA (1x) for a maximum of 6 min at 37 °C. Culture medium was added in a 1:1 ratio to the trypsin-EDTA and the cell suspension was centrifuged at 700 rpm for 3 min at 37 °C before the pellet was resuspended in 37 °C culture medium. 1.0 × 10^5^ cells in 100 µL was seeded onto transparent polyethylene terephthalate membrane inserts (ThinCert™, pore size: 1 μm, area: 33.6 mm^2^) (Greiner Bio-One, Frickenhausen, Germany) in a Transwell setup (Cellstar^®^, Greiner Bio-One, Frickenhausen, Germany) with 260 µL culture medium in the basolateral chamber. After 24 h the apical medium was removed, and the cells were cultured under air-liquid interface (ALI) conditions for 20–21 days. Every second day, the cells were washed apically with PBS and the basolateral medium was changed.

**Table 1:** Media for extraction and cultivation of olfactory epihtelial cells

Pronase medium	EEBS, 1.4 mg/mL pronase, 0.2 U/mL penicillin, 0.2 µg/mL streptomycin, 0.2 mg/mL gentamycin sulphate, 0.2 mg/mL kanamycin sulphate, 5 µg/mL amphotericin B
Adhesion medium	DMEM F-12, 20% FBS, 2 mM glutamine, 4.5 mg/mL glucose, 1% MEM NEAA, 0.2 u/mL penicillin 0.2 µg/mL streptomycin, 0.2 mg/mL gentamycin sulphate, 0.2 mg/mL kanamycin sulphate, 5 µg/mL amphotericin B
Cultivation medium	DMEM F-12, 10% FBS, 2 mM glutamine, 4.5 mg/mL glucose, 1% MEM NEAA, 0.2 u/mL penicillin 0.2 µg/mL streptomycin, 0.2 mg/mL gentamycin sulphate, 0.2 mg/mL kanamycin sulphate, 5 µg/mL amphotericin B

EEBS: Earle’s balanced salt solution; DMEM: Dulbecco’s Modified Eagle Medium; FBS: Fetal bovine serum; NEAA: Non-essential amino acids

### Dot blotting

Samples were collected from washing the olfactory epithelial cells in culture flasks or on transwells with 1 mL PBS and stored at -20 °C until use. 40 µL samples were applied to a wet polyvinylidene difluoride membrane (Bio-Rad, Hercules, CA, USA), which was subsequently incubated in blocking solution (5% (w/v) skimmed milk powder in TPBS (0.1% (v/v) Tween^®^ 20 in PBS)) for 2 h on a shaking table at room temperature (RT). The membrane was incubated with an anti-MUC5AC primary antibody (1:2,500 in blocking solution) (NBP2-15196, Novus Biologicals, Centennial, CO, USA) for 24 h at 4 °C and thereafter washed three times with TPBS. Subsequently, the membrane was incubated with horse radish peroxidase-conjugated goat-anti-mouse antibody (1:4,000) for 1 h at RT before washing three times with TPBS. The membrane was incubated with ECL™ Prime Western Blotting Detection Reagent for 5 min at RT and imaged using a FluorChem Q imaging system (Alpha Innotech, Santa Clara, CA, USA).

### Immunocytochemistry

Cells cultured under ALI conditions for 21 days were immuno-stained for the proteins listed in Table 2. The cells were washed with three times with PBS and fixated by adding 4% paraformaldehyde for 15 min before being permeabilized with 0.1% Triton X-100 in blocking solution (2% BSA in PBS) for 5 min at room temperature. The cells were washed three times with PBS and thereafter incubated for 30 min in blocking solution at room temperature, followed by incubation with primary antibodies (Table 2) in blocking solution at 4°C overnight. The cells were washed three times with PBS and incubated with 4’,6-diamidino-2-phenylindole (DAPI) (1:1,000) and Alexa Fluor 488-conjugated secondary antibodies (1:300) (goat-anti-rabbit or goat-anti-mouse (Thermo Fischer Scientific, Dreieich, Germany)), in blocking solution for 30 min at room temperature. The cells were washed three times with PBS before being transferred to an object slide and mounted in mounting solution (Shandon Immu-Mount, Thermo Fischer Scientific, Roskilde, Denmark). Samples were imaged using a fluorescence microscope (zonular occludens-1, claudin-1, claudin-5) (Leica Microsystems at Danaher, Wetzlar, Germany) or a Zeiss LSM 710 laser scanning microscope (aquaporin 5, acetylated tubulin, occludin, claudin-3, claudin-4) (Carl Zeiss, Jena, Germany).

**Table 2:** Overview of primary antibodies used for immunocytochemistry

Protein	Cat. no.	Supplier	Host species	Dilution
Aquaporin 5	PA5-36529	Thermo Fischer Scientific	Rabbit	1:100
Acetylated tubulin	T7451	Merck KGaA	Mouse	1:600
Occludin	711,500	Thermo Fischer Scientific	Rabbit	1:100
Zonula occludens-1	61-7300	Thermo Fischer Scientific	Rabbit	1:100
Claudin-1	NBP1-77036	Novus Biologicals	Rabbit	1:50
Claudin-3	PA5-32353	Thermo Fischer Scientific	Rabbit	1:100
Claudin-4	Ab53156	Abcam	Rabbit	1:200
Claudin-5	Ab15106	Abcam	Rabbit	1:100

### Cellular metabolic activity

The cellular metabolic activity was assessed on olfactory epithelial cells cultured in a 96-well plate for 48 h using a commercially available sodium 3´-[1-(phenylaminocarbonyl)-3,4-tetrazolium]-bis (4-methoxy-6-nitro) benzene sulfonic acid hydrate (XTT) assay as directed by the manufacturer. The olfactory epithelial cells were subjected to 4 h incubation (37 °C, 5% CO_2_, 95% relative humidity) with 50 or 100 µM Tat, LowPro, or PenShuf in transport medium. Cells incubated with transport medium (MEM w/o phenol red, 0.05% BSA, 0.1 U/mL Penicillin, 0.1 µg/mL Streptomycin) without peptides served as live cell controls and wells with transport medium but no cells served as dead cell controls. After peptide incubation, the cells were gently washed twice with transport medium before incubation with 100 µL transport medium and 50 µL XTT labelling mixture (electron-coupling reagent: XTT labelling reagent 1:50) for 1 h at 37 °C with horizontal shaking (90 rpm). Samples (100 µL) from each well were transferred to a new 96-well plate and the absorbance was measured at 466 nm using a SPECTROstar Nano plate reader (BMG Labtech, Ortenberg, Germany). The relative cellular viability was calculated by Eq. 1:

1 $$\:Viable\:cells\:\left(\%\right)=\:\frac{{A}_{sample}-{A}_{dead\:cell\:control}}{{A}_{live\:cell\:control}-{A}_{dead\:cell\:control}}*\:100\%\:\:\:\:$$

### Transepithelial electrical resistance

The transepithelial electrical resistance (TEER) was measured across the olfactory epithelial cell monolayers cultured for 20 or 21 days under ALI conditions. 300 µL transport medium (MEM w/o phenol red, 0.05% BSA, 0.1 U/mL Penicillin, 0.1 µg/mL Streptomycin) or transport buffer (10 mM HEPES, 0.05% BSA in Hanks Balanced Salt Solution (HBSS), pH 7.4) was added to the basolateral chamber and the cells were allowed to equilibrate for 15 min at 37 °C, 5% CO2, 95% relative humidity). Thereafter, the cells were equilibrated to RT prior measuring the TEER with a chopstick electrode or in an Endohm-24 cup connected to an Epithelial Volt/Ohm meter (both World Precision Instruments, Sarasota, FL, USA). The measured TEER values were subtracted the resistance across an empty Transwell insert and multiplied with the area of the insert (0.33 cm^2^) to obtain the standardized unit Ω⋅cm^2^.

### Transport studies

Transport studies were performed on olfactory epithelial cell monolayers cultured for 20 or 21 days under ALI conditions. The experiments with the marker molecules sodium fluorescein and 4 KDa fluorescein isothiocyanate (FITC) dextran were initiated by gently applying 300 µL samples (both compounds 0.5 mg/mL) in 37 °C transport medium to the apical compartment. 50 µL samples were withdrawn at time points 0.5, 1, 1.5, 2, 3, 4, and 24 h into black clear-bottom 96-well plates. The sampling volume was immediately replaced with 37 °C transport medium. For analysis, the fluorescence was measured using an Infinite M Plex fluorescence plate reader (TECAN, Männedorf, Switzerland) with excitation/emission set to 460/515 nm for sodium fluorescein and 490/520 nm for FITC-dextran. The experiments with NA-1 and the cell-penetrating peptides were initiated by gently applying 300 µL samples (50 µM NA-1 alone or co-administered with 50 µM Tat, LowPro, or PenShuf) in 37 °C transport buffer to the apical compartment. 75 µL samples were withdrawn at time points 1, 2, 3, and 16 h into glass vials. The sampling volume was immediately replaced with 37 °C transport buffer. NA-1 was labelled with selenomethionine (M^Se^) between the Tat and the NR2B9c moiety to facilitate analysis through selenium quantification using inductively coupled plasma mass spectrometry (ICP-MS). For selenium analysis, the samples were subjected to flow injection analysis on a Dionex Ultimate 3000 UPLC (Thermo Fischer Scientific, Germering, Germany) with ICP-MS detection using an Agilent 8800 Triple Quad equipped with a 2.5 mm quartz torch with platinum sampler -and skimmer cones (Agilent Technologies, Santa Clara, CA, USA). The mobile phase was 5% methanol and 0.1% trifluoroacetic acid in ultrapure water. 10 µL samples were introduced with a flow rate of 0.3 mL/min via a Micromist U-series nebulizer (Glass Expansion, Melbourne, Australia) and a double-pass concentric spray chamber (Agilent Technologies, Santa Clara, USA). The plasma gas flow was set to 15 mL/min. Oxygen was applied as reaction gas. ICP-MS operating conditions are summarized in Table 3. Daily optimization was performed using a 100 µg/L Se solution (1000 ± 6 mg/L, SCP Science, Canada) diluted in mobile phase. A standard curve was prepared on every measurement day. Lower limit of detection was 0.14 µg/mL and lower limit of quantification was 0.41 µg/mL.

**Table 3:** Operating conditions for inductively coupled plasma mass spectrometry

Plasma gas	Argon
Reaction gas	Oxygen (O_2_)
Plasma gas flow	15 mL/min
Flow rate	0.300 µL/min
Injection volume	10 µL
Run time	0.5 min
Needle wash before injection (mobile phase)	50 µL
Isotopes Q_1_ → Q_2_	^80^Se → ^96^SeO

The apparent permeability (P_app_) coefficients for sodium fluorescein, FITC-dextran, and NA-1 was calculated by Eq. 2:

2 $$\:P_{app}\:(cm/s)=\:\frac{dQ}{dt}*\:\frac{1}{A*C0}\:\:\:\:$$

Where *dQ/dt* is the steady state flux, *A* is the area of the filter insert (0.33 cm^2^), and *C*0 is the donor concentration in the apical chamber at time-point 0 min.

### Biodistribution

9–12 week old female NMRI mice (Envigo RMS B.V, Venray, the Netherlands) were deeply anesthetized by subcutaneous injection with 0.1 mL/10 g body weight Hypnorm/Dormicum (fentanyl/fluanisone: midazolam: sterile water 1:1:2) before nasal administration of sodium fluorescein, 4 KDa FITC-dextran, or NA-1 (alone or co-administered with Tat, LowPro, or PenShuf in a 1:1 molar ratio). For each of the test compounds, 3 nmol/g body weight were administered in 4 µL 0.9% NaCl per nostril using a 200 µL natural capillary tip (Qualitix^®^ Superior Pipette Tips, Socorex Isba SA, Switzerland) inserted approximately 10 mm into each nostril directed against the olfactory mucosa. In an intravenous reference group, NA-1 was administered as a bolus injection (3 nmol/g body weight in 0.9% NaCl) into the tail vein. After 60 min compound circulation, the animals were transcardially perfused with 0.01 M PBS (pH 7.4). The brain, olfactory bulb, heart, lungs, spleen, kidneys, liver, and 2–3 cm of the upper part of duodenum were collected in 2 mL tubes and stored at -70 °C until analysis. Biodistribution of sodium fluorescein and 3–5 KDa FITC-dextran was analyzed by fluorescence measurements. The tissues were homogenized in 0.15-1 mL ice-cold lysis buffer (10 mM Tris-HCl, 0.25 M sucrose, 1 mM ethylenediaminetetraacetic acid, 1 mM ethylene glycol-bis-(beta-aminoethylether)-N, N,N’,N’-tetraacetic acid, 2% Tergitol™) using a 7 mL KIMBLE^®^ dounce tissue grinder (Merck KGaA, Darmstadt, Germany). The homogenate was spun down for 10 min at 14,000 rpm and 4 °C in a Sigma 1–15 K centrifuge (DJB Labcare, Newport Pagnell, England). 50 µL supernatant was transferred to a 96-well black clear-bottom plate and the fluorescence was measured using a NOVOStar fluorescene plate reader (BMG Labtech, Ortenberg, Germany) with excitation/emission set to 485/590 nm. Quantification was performed using calibration curves for each compound in homogenate of the respective tissues. Biodistribution of NA-1 was assessed by measuring the selenium content in tissue digested in HNO_3_ for 3–5 days at room temperature (50 mg tissue per 0.5 mL HNO_3_ for heart, lungs, spleen, kidney, and duodenum or 0.2 mL HNO_3_ for olfactory bulb and 1 mL HNO_3_ for the remaining brain. The tissue samples were spun down for 5 min at 10,000 rpm in a Sigma 1–15 K centrifuge (DJB Labcare, Newport Pagnell, England) before dilution (1:5–1:32) in 5% methanol with 0.1% trifluoroacetic acid in ultrapure water. The samples were analyzed with ICP-MS as described for *transport studies*, but with a 20 µL injection volume and a 2 min run time.

### Data and statistical analysis

Microsoft Office Excel v2016 (Microsoft, Redmont, WA, USA) was used for data processing and GraphPad Prism v9 (GraphPad, La Jolla, CA, USA) for data presentation and statistical analyses. Data are presented as mean ± standard deviation (SD) with N and n representing the number of technical -and biological replicates, respectively. Unpaired -or paired t-test or one-way -or two-way analysis of variance (ANOVA) with Tukey’s -or Dunnett’s multiple comparisons test were applied. Data with p-values < 0.5 were considered statistically significant.

## Results and discussion

*Primary model of the olfactory mucosa displayed mucus production and epithelial barrier properties; and thus suitability for permeation studies*.

**Fig. 1:**
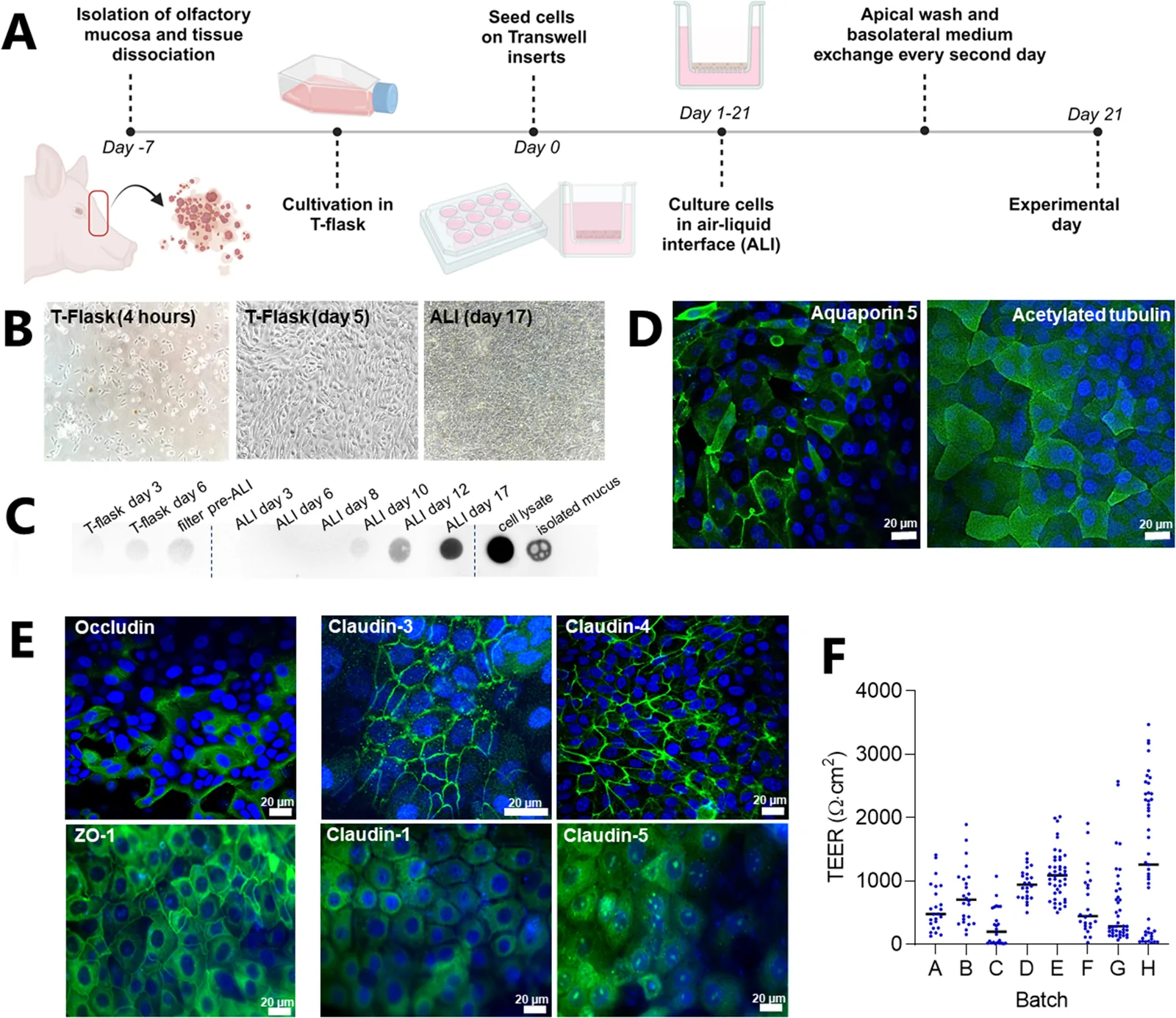
Setting up the primary porcine olfactory epithelial model. (**A**) Overview of protocol. (**B**) Representative light microscopy images of primary porcine olfactory epithelial cells grown in T-flask or on a transwell insert in air-liquid interface. (**C**) Mucus production at different cultivation days evaluated by dot blotting. (**D**) Representative fluorescence microscopy immunostaining images of aquaporin 5 (green, left) and acetylated tubilin (green, right) in primary porcine olfactory epithelial monolayers. Blue = nucleus staining, scale bars = 20 μm. (**E**) Representative fluorescence microscopy immunostaining images of ollcudin, zonula occludens-1 (ZO-1), claudin-1, -3, -4, and 5 (green) in primary porcine olfactory epithelial monolayers. Blue = nucleus staining, scale bars = 20 μm. (**F**) Transepithelial electrical resistance (TEER) measurements across batches (A-H) of primary porcine olfactory epithelial monolayers measured on day 21. Data are presented as single datapoints with median values (*N* = 24, *n* = 3)

A state-of-the art primary porcine in vitro model was set up according to Ladel et al. [14] (Fig. 1) in order to facilitate initial in vitro studies to evaluate potential adverse effects on the mucosal barrier upon incubation with NA-1 and the cell-penetrating peptides as well as the ability of NA-1 to traverse the barrier. The olfactory mucosa was isolated from snouts of 2–4 month-old pigs and cultured in T-flasks prior seeding on Transwell^®^ inserts upon which they were allowed to mature and polarize in an ALI (Fig. 1A, B).

The presence of mucus and cilia are important features for a functional olfactory mucosa and may affect drug clearance from the nasal cavity [15]. Dot blots with an antibody directed against mucin-4 revealed some mucus production already during T-flask cultivation (Fig. 1C). The mucus production was reset upon ALI cultivation on transwell inserts, but reestablished and gradually increased with culture time. We moreover, confirmed the expression of aquaporin 5 using immunostaining (Fig. 1D, left), since aquaporin 5 is important for normal mucus secretion from the Bowman’s gland [16]. Cilia production was confirmed by immunostaining of acetylated tubulin (Fig. 1D, right). The tight junction proteins occludin and claudin provide tightness to epithelial barriers and are therefore important for an in vitro model to allow drug transport studies. We observed the presence of occludin and the scaffolding protein ZO-1 as wells as the claudin isoforms 1, 3, 4, and 5 (Fig. 1E), which is in agreement with Steinke et al. [16], revealing the molecular composition of tight junctions in the rat olfactory epithelium. Our immunostainings documented that claudin-3 -and 4 appeared more localized to the intercellular zones, when compared to claudin-1 -and 5, thus suggesting that the former provides the barrier property in the model. This observation aligns well with a recent study demonstrating that claudin-3 expression appeared proportional to the tightness of human nasal epithelial cells in vitro [17]. We assessed the barrier integrity through TEER measurements, revealing some intra-batch and batch-to-batch variation, but with an average TEER of 793 Ω x cm^2^ (Fig. 1F), which matches values reported by Ladel et al. with an average of 648 Ω x cm^2^ [14].

*Model compounds display size-dependent permeation across the primary olfactory mucosa in vitro model*,* whereas size-dependent uptake of compounds in vivo is questioned*.

**Fig. 2:**
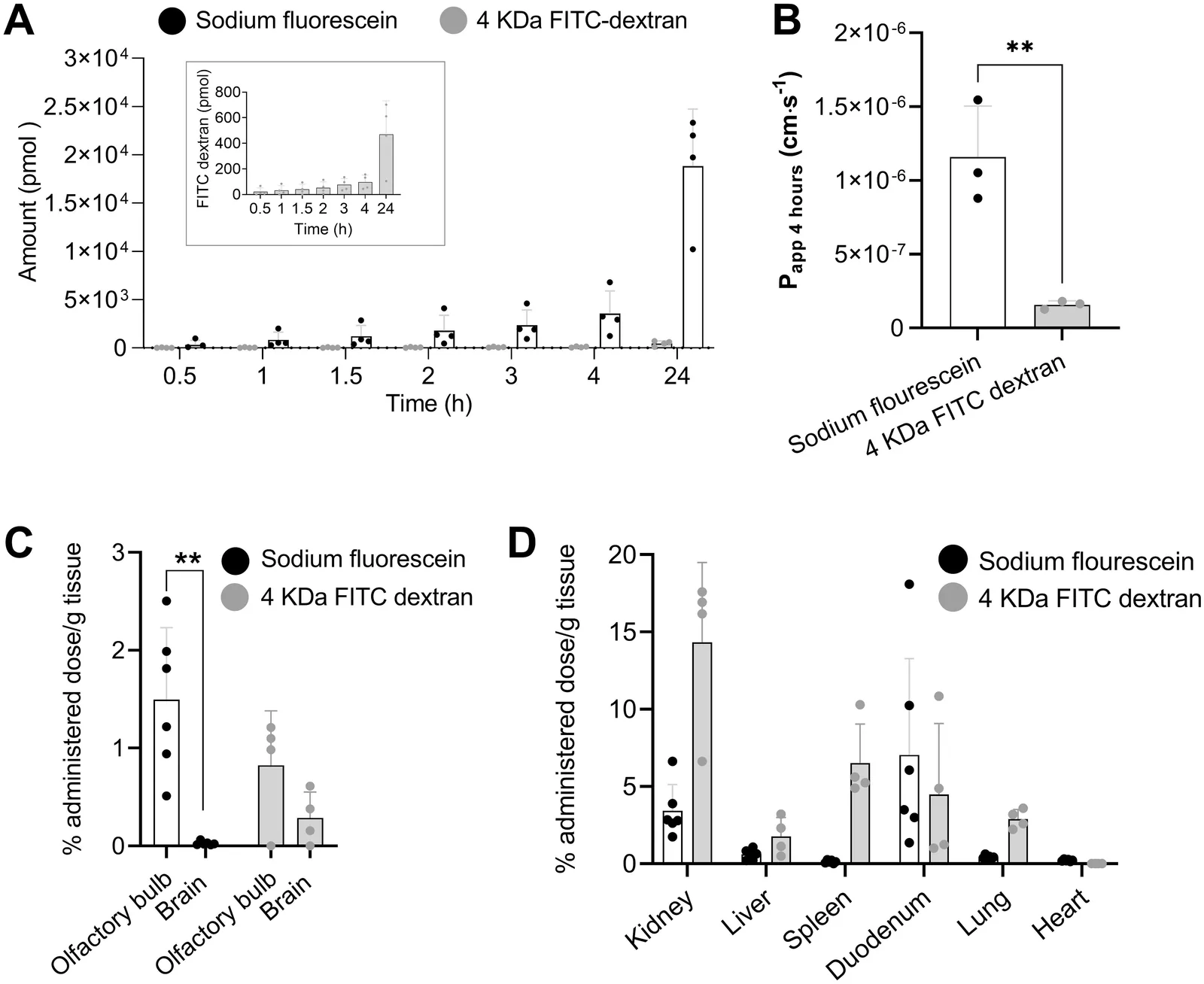
Transepitelial in vitro transport -and brain uptake in mice upon nasal administration of markers compounds. (**A**) Sodium fluorescein (376 g/mol) and FITC-dextran (4000 g/mol) transport across primary porcine olfactory epithelial monolayers over 24 h. Data are presented as mean + SD (*N* = 3–4, *n* = 3). (**B**) Apparent permeabilities (P_app_) for sodium fluorescein and FITC-dextran across primary porcine olfactory monolayers calculated from flux curves from 0.5–4 h. Data are presented as mean + SD (*N* = 3, *n* = 3); **: *p* < 0.01 (paired t-test). (**C**) Distribution of sodium fluorescein and FITC-dextran in the olfactory bulb and brain of mice 1 h after nasal administration. Data are presented as mean + SD (*N* = 4–6); **: *p* < 0.01 (unpaired t-test). (**D**) Off-target tissue of sodium fluorescein and FITC-dextran in mice 1 h after nasal administration. Data are presented as mean + SD (*N* = 4–6)

To further characterize the barrier functionality of our olfactory mucosa in vitro model, we investigated the permeation of sodium fluorescein (376 g/mol) and FITC-dextran (4,000 g/mol), representing a small -and a peptide-sized compound, respectively (Fig. 2A, B). Upon 24 h of incubation 29.3% and 3.7% of the applied sodium fluorescein and FITC-dextran, respectively, had permeated the barrier, thus demonstrating size-selectivity of the model (Fig. 2A). The apparent permeabilities were calculated to the 4 h time point, since sink conditions were not maintained for sodium fluorescein at the 24 h time point (Fig. 2B). These revealed a 7-fold difference when comparing the permeabilities for sodium fluorescein (1.16 × 10^− 6^ cm/sec) and FITC-dextran (0.16 × 10^− 6^ cm/sec), aligning well with reported values by Ladel et al. [14]. Next, we investigated whether a similar size-selectivity was observed upon nasal administration to mice (Fig. 2C, D). Sodium fluorescein, and to some extent FITC-dextran, accumulated in the olfactory bulb to a greater extent when compared to the brain (Fig. 2C). However, we did not observe the same size-selectivity, as observed in vitro, when comparing the accumulation of sodium fluorescein with the accumulation of FITC-dextran in the olfactory bulb. In contrast, there was a tendency toward greater brain uptake of FITC-dextran when compared to the brain uptake of sodium fluorescein. In addition, FITC-dextran appeared to accumulate in the liver, spleen, and lungs to a greater extent than sodium fluorescein (Fig. 2D). This may be due to more pronounced systemic uptake of FITC-dextran through the respiratory epithelium and the peripheral blood flow, and/or faster systemic clearance of sodium fluorescein when compared to FITC-dextran.

*NA-1 permeated the in vitro olfactory mucosa model*,* whereas co-administration with PenShuf improved NA-1 permeation*,* but also resulted in some adverse barrier effects*.

Nasal administration to the olfactory mucosa may be relevant for brain delivery of NA-1, and co-administration with selected cell-penetrating peptides may boost the brain uptake of NA-1. Prior advancing to in vivo testing, we evaluated potential adverse effects on the olfactory mucosa in vitro model of the cell-penetrating peptides as well as the permeation potential of NA-1. Selenomethionine was introduced to the NA-1 sequence (Table 2) to allow sensitive detection in the nanomolar range using ICP-MS [18]. Tat, LowPro, and PenShuf were selected as relevant cell-penetrating peptides due to their demonstrated potential as peptide shuttles for therapeutic peptide delivery across the olfactory mucosa [12, 13]. NA-1 and the cell-penetrating peptides all bear a net positive charge at physiological pH (Table 2).

**Table 2:** Primary sequences, molecular weight (Mw), and net charge at pH 7.4 for NA-1 and the cell-penetrating peptides Tat, LowPro, and PenShuf. The Tat-moiety of the NA-1 sequence is composed of D-amino acids and selenomethionine (M^Se^) is introduced into the sequence to allow quantitative analysis using inductively coupled plasma mass spectromerty

Name	Sequence	Mw (g/mol)	Net charge at pH 7.4
NA-1	ygrkkrrqrrr-M^Se^-KLSSIESDV	2696.86	+ 8
Tat	YGRKKRRQRRR	1559.82	+ 8
LowPro	VSRRRRRRGGRRRR	1878.23	+ 10
PenShuf	RWFKIQMQIRRWKNKK	2245.79	+ 7

**Fig. 3:**
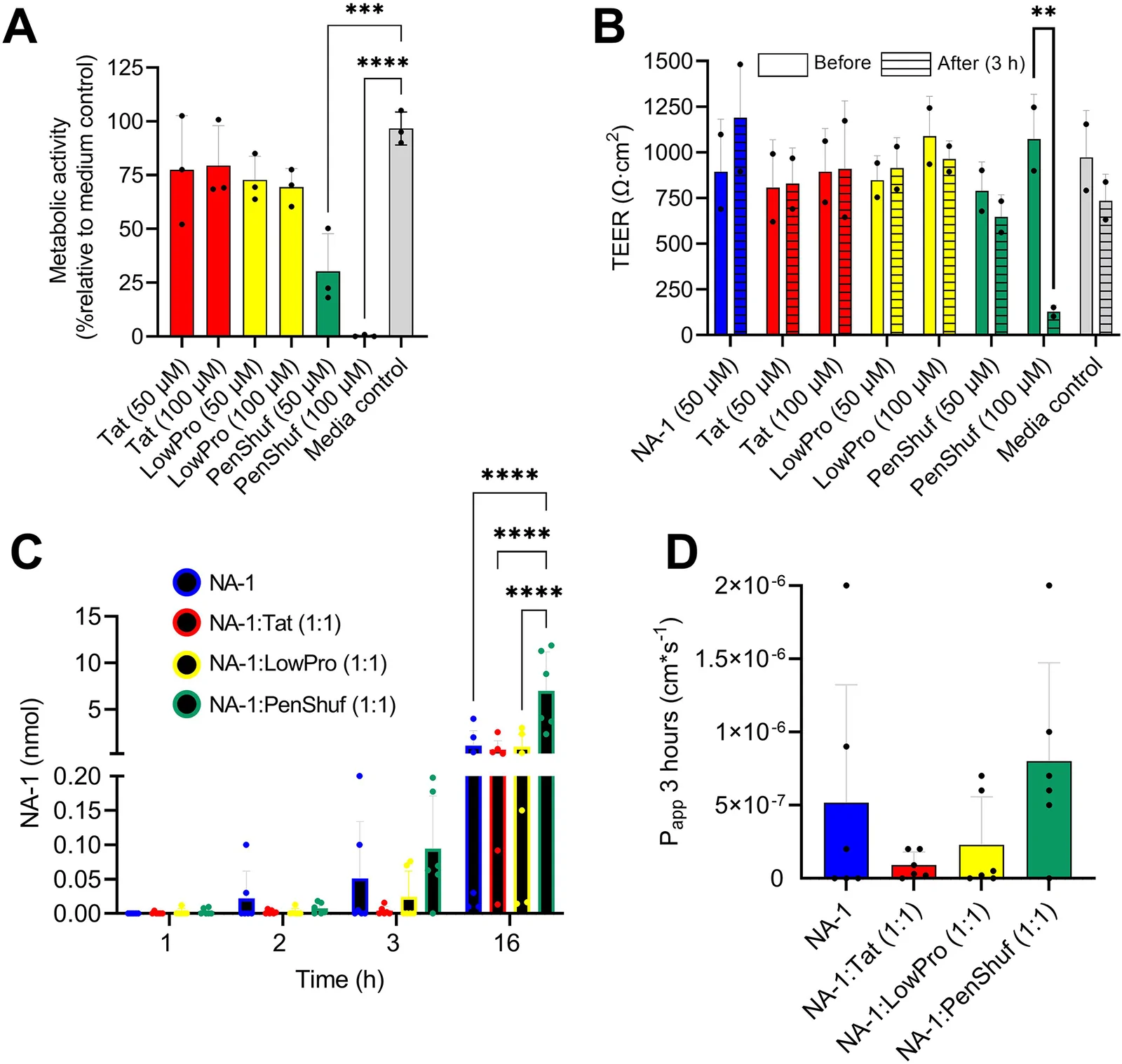
Metabolic activity of porcine primary olfactory epithelial cells and barrier integrity and NA-1 transport across primary porcine olfactory epithelial monolayers. (**A**) Cellular metabolic activiy presented as percentage reletive to cell media control upon incubation with 50 or 100 µM Tat, LowPro, or PenShuf. Data are presented as mean + SD (*N* = 3, *n* = 3); ***: *p* < 0.001, ****: *p* < 0.0001 (one-way ANOVA). (**B**) Transepithelial electrical resistance (TEER) measurements across primary porcine olfactory epithelial monolayers before and after 3 h incubation with 50 µM NA-1 or 50 or 100 µM Tat, LowPro, or PenShuf. Data are presented as mean + SD (*N* = 3, *n* = 2); **: *p* < 0.01 (one-way ANOVA). Transport of 50 µM NA-1 applied alone or co-administered with 50 µM Tat, LowPro, or PenShuf across primary porcine olfactory epithelial monolayers over 16 h. Data are presented as mean + SD (*N* = 6); ****: *p* < 0.0001 (one-way ANOVA). (**D**) Apparent permeabilities (P_app_) 50 µM NA-1 applied alone or co-administered with 50 µM Tat, LowPro, or PenShuf across primary porcine olfactory monolayers calculated from flux curves from 1–3 h. Data are presented as mean + SD (*N* = 6)

First, we evaluated potential adverse effects on the primary olfactory mucosa cells upon incubation with Tat, LowPro, or PenShuf (Fig. 3A). A slight decrease in the cellular metabolic activity was observed, relative to cell culture media controls, upon incubation with 50 or 100 µM Tat or LowPro, whereas a marked concentration-dependent reduction in the cellular metabolic activity was observed upon incubation with PenShuf. When comparing the barrier resistance in terms of measured TEER before and after incubation with NA-1, Tat, LowPro, or PenShuf, PenShuf appeared to negatively affect the barrier integrity, but only in the highest applied concentration (100 µM) (Fig. 3B). In agreement with that observation, Berthelsen et al. recently demonstrated that PenShuf negatively affects the cellular metabolic activity as well as epithelial monolayer integrity when applied to the human intestinal epithelial Caco-2 cell culture model [19]. PenShuf, but not Tat and LowPro, contains two tryptophan residues in its sequence. The presence and specific positioning of tryptophan in arginine-rich cell-penetrating peptides have been suggested to promote interaction with glycosaminoglycans presented at epithelial cell surfaces [20], which in some concentrations apparently leads to adverse cellular effects as demonstrated in this study (Fig. 3A, B). Next, we applied NA-1 alone or co-administered in a 1:1 molar ratio with Tat, LowPro, or PenShuf to the primary olfactory mucosa model for 16 h incubation and sampling at time-points 1, 2, 3, and 16 h (Fig. 3C). Within the first 3 h, NA-1 appeared to permeate the barrier more efficiently alone, when compared to being co-administered with Tat and LowPro, whereas co-administration with PenShuf significantly improved the barrier permeation of NA-1 at the 16 h time-point. However, the effect exerted by PenShuf is likely a cause of adverse effects on the cellular metabolic activity (Fig. 3A) resulting in a compromised barrier (Fig. 3B), rather than solely acting through a reversible mechanism leaving the barrier intact. However, the PenShuf facilitated NA-1 permeation was also reflected in the calculated permeabilities for the 3 h time-point (P_app NA−1_ = 0.51 × 10^− 6^ cm/sec vs. P_app NA−1:PenShuf_. = 0.80 × 10^− 6^ cm/sec) (Fig. 3D), at which PenShuf did not affect the measured TEER (Fig. 3B). Tat and LowPro did not improve the permeability of NA-1 (P_app NA−1:Tat_ 0.09 × 10^− 6^ cm/sec; P_app NA−1:LowPro_ 0.22 × 10^− 6^ cm/sec). Nevertheless, NA-1 by itself displayed a 3.2-fold better permeation potential, when compared to the 4 KDa FITC-dextran (Fig. 2B), which in this context can be considered as a similar sized compound primarily permeating the barrier by passive paracellular diffusion. In contrast, is the build-in Tat moiety in the NA-1 sequence (Table 2) responsible for facilitating endocytosis of NA-1, potentially followed by transcytosis, as earlier demonstrated when assessing blood-brain barrier permeation in vitro [6] and brain uptake in vivo [7] of Tat-conjugated NR2B9c (i.e. NA-1) vs. NR2B9c without the Tat moiety.

*NA-1 is delivered to the brain upon nasal administration with uptake into the olfactory bulb improved by co-administration with the cell-penetrating peptides LowPro and Tat*.

**Fig. 4:**
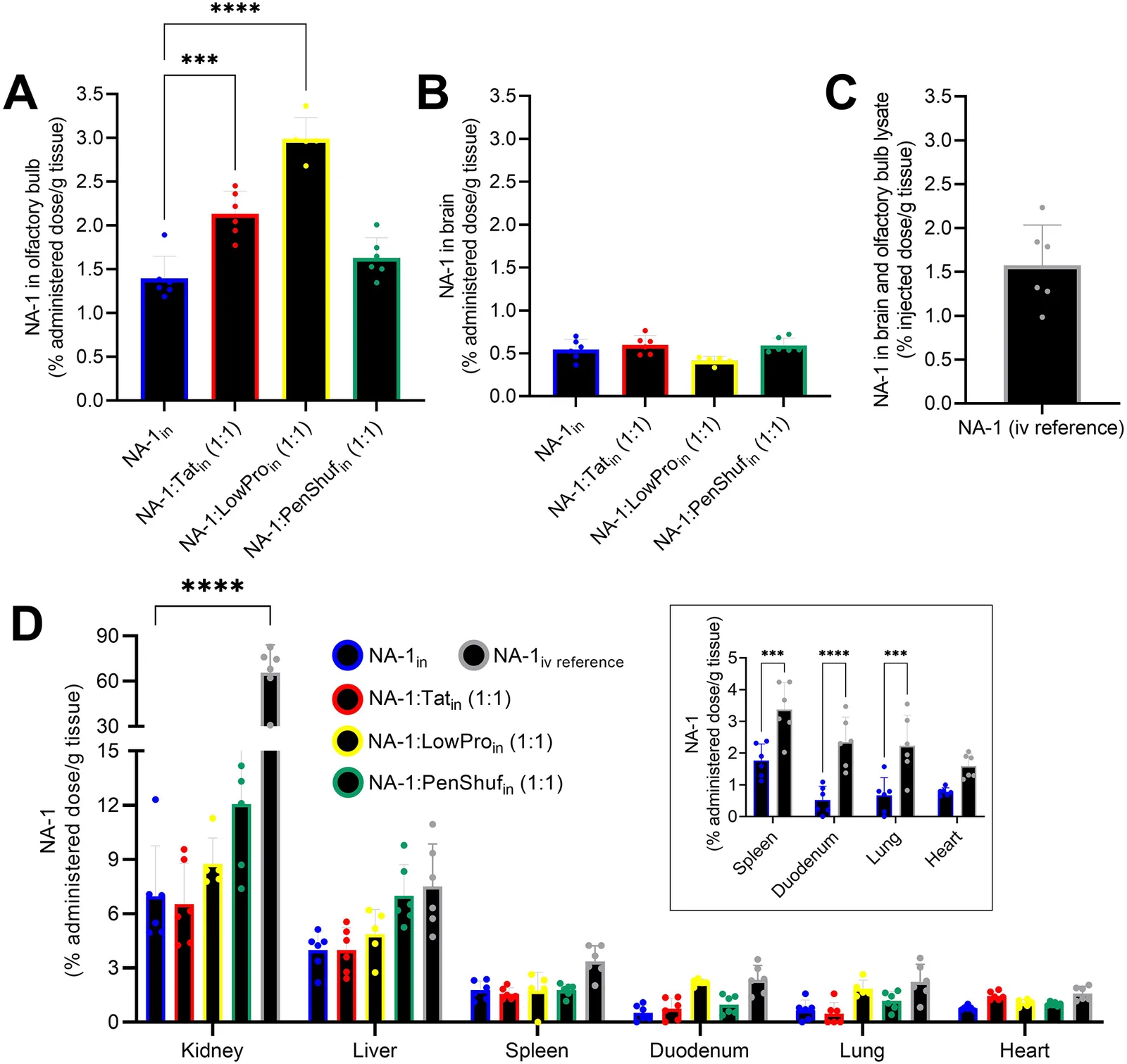
Biodistribution of NA-1 administered alone or co-adminitered with Tat or LowPro. (**A**) Distribution of NA-1 in the olfactory bulb and (**B**) brain of mice 1 h after nasal administration, or (**C**) NA-1 in whole brain lysate 1 h after intravenous administration. Data are presented as mean + SD (*N* = 4–6); ***: *p* < 0.001, ****: *p* < 0.0001 (one-way ANOVA). (**D**) Off-target tissue of NA-1 in mice 1 h after nasal administration. Data are presented as mean + SD (*N* = 4–6 ); ***: *p* < 0.001, ****: *p* < 0.0001 (two-way ANOVA)

Finally, we investigated whether direct nose-to-brain delivery of NA-1 is obtained upon administration to the upper nasal cavity (i.e. the olfactory mucosa), and whether co-administration with cell-penetrating peptides may boost NA-1 delivery to the brain (Fig. 4). Mice were subjected to nasal administration of NA-1 alone or mixed in a 1:1 molar ratio with Tat, LowPro, or PenShuf. NA-1 was quantified in the olfactory bulb (Fig. 4A) and the remaining brain (Fig. 4B) 1 h after administration with intravenously administered NA-1 serving as reference (Fig. 4C). NA-1 off-tissue distribution was evaluated in the kidney, liver, spleen, duodenum, lung, and heart (Fig. 4D). We observed a larger degree of NA-1 accumulation in the olfactory bulb (1.38% administered dose/g) when compared to the remaining brain (0.54% administered dose/g) (Fig. 4A, B), and that to a greater extent when NA-1 was co-administered with Tat (2.13% administered dose/g) or LowPro (2.98% administered dose/g) but not with PenShuf (1.63% administered dose/g). Neither of the cell-penetrating peptides improved NA-1 accumulation in the brain as observed in the olfactory bulb, and the more pronounced effect of co-administration with PenShuf observed in vitro (Fig. 3C, D) was not translated in vivo. The intravenously dosed NA-1 control reached 1.57% injected dose/g, when analyzed in whole brain lysate, which includes the olfactory bulb and the remaining brain (Fig. 4C). This is notably more when compared to earlier studies in mice (0.3% injected dose/g) [6] and rats (0.08% injected dose/g) [7] receiving a similar intravenous dose of NA-1 composed of all-L-amino acids and conjugated to the fluorophore TAMRA to allow detection. In contrast, the NA-1 included in the present study contains a Tat moiety composed of the more enzyme resilient D-amino acids and the smaller selenomethionine for analytical purposes. However, combining the amount of NA-1 reaching the olfactory bulb and the brain upon nasal administration 1.92% of administered dose/g was detected, which is of similar magnitude as the intravenous control (*p* = 0.15, unpaired t-test). In addition, NA-1 detected in the whole brain lysates of the intravenous control group reflects both peptide adhering to the surface of the brain vasculature and peptide internalized into the vascular endothelial cells in addition to peptide reaching brain parenchyma [7]; and thus not necessarily NA-1 available for neuronal uptake and target engagement. In contrast, NA-1 detected in the olfactory bulb and brain upon nasal administration (Fig. 4A, B) has presumably entered the parenchyma either via the olfactory mucosa, facilitating uptake through the olfactory bulb, or through the trigeminal nerve endings in the respiratory epithelium leading to more posterior brain uptake. Finally, we evaluated off-tissue distribution of the NA-1 intravenous control or NA-1 upon nasal administration alone or as co-administered with Tat, LowPro, or PenShuf (Fig. 4D). Nasal administered NA-1 was observed in all off-target tissues (kidney > liver > spleen > duodenum > lung > heart), whereas this distribution was not affected by co-administration with the cell-penetrating peptides. This suggests that a fraction of the nasally administered NA-1 enters systemic circulation through absorption across the respiratory epithelium, which is difficult to avoid given the small size of the nasal cavity in mice. A small fraction of the administered peptide solution may also be swallowed and thus give additional rise to peptide accumulation in the duodenum. However, a significantly higher amount of NA-1 was detected in the kidneys (9.5 fold), spleen (1.6 fold), duodenum (4.5 fold), and lungs (2.8 fold) upon intravenous administration when compared to nasal administration (Fig. 4D).

## Conclusion

We have demonstrated that NA-1 is delivered to the brain upon nasal administration to mice and that nasal administration is associated with significantly less off-tissue distribution when compared to intravenously administered NA-1. Co-administration with the cell-penetrating peptides Tat or LowPro improved NA-1 delivery into the olfactory bulb, but not to the remaining brain, thus questioning their relevance in drug delivery to posterior brain regions. However, whether increasing the molar ratio between NA-1 and the cell-penetrating peptides may represents a strategy to improve NA-1 delivery to brain regions beyond the olfactory bulb remains unknown. Nevertheless, nasal administration of NA-1 may represent viable alternative to systemic administration to allow parallel pharmacological treatment of excitotoxicity and thrombolysis with the blood clot resolving compound alteplase during the acute phase of ischemic stroke.

## Data Availability

The data generated for the current study are available from the corresponding author on reasonable request.
